# Psychological mechanisms connected to dissociation: Generating hypotheses using network analyses

**DOI:** 10.1016/j.jpsychires.2022.01.049

**Published:** 2022-04

**Authors:** Emma Černis, Anke Ehlers, Daniel Freeman

**Affiliations:** a University of Oxford Department of Psychiatry, Warneford Hospital, Oxford, OX3 7JX, UK; b Oxford Centre for Anxiety Disorders and Trauma, Department of Experimental Psychology, University of Oxford, The Old Rectory, Paradise Square, Oxford, OX1 1TW, UK; c Oxford Health NHS Foundation Trust, Warneford Hospital, Oxford, OX3 7JX, UK

**Keywords:** Dissociation, Network analysis, Perseverative thinking, Self-efficacy, Psychological mechanisms

## Abstract

A large number of mechanisms, many relating to the processing of affect, have been proposed to cause dissociation. The aim of this study was to use network analyses to identify psychological processes most closely connected with ‘felt sense of anomaly’ dissociative experiences. Both an undirected model and a partially directed network model were estimated using data from 6161 general population respondents collected online. The networks were used to identify relationships between dissociation and ten candidate mechanisms: cognitive appraisals, behavioural responses to dissociation, affect intolerance, alexithymia, attentional control, body vigilance, anxiety sensitivity, general self-efficacy, perseverative thinking, and beliefs regarding stress. Both models indicated a highly connected network in which dissociation had direct connections with six psychological processes: cognitive appraisals, behavioural responses, perseverative thinking, alexithymia, general self-efficacy, and beliefs about being overwhelmed. The strongest connection in both networks was between dissociation and cognitive appraisals (causal effect 0.73). The causal direction of connections could not be statistically determined with confidence, apart from the strong probability that dissociation causes meta-cognitions about being overwhelmed (98.54% of 50,000 sampled directed acyclic graphs). Both networks suggest that cognitive appraisals and factors relating to heightened (negative) sensitivity to affect are closely connected to dissociation. Dissociative experiences may arise from a high sensitivity to affect leading to threat-based appraisals that are ruminated upon and maintained by unhelpful behaviours such as avoidance. Investigation of these relationships in clinical groups, and direct causal tests, are required.

## Highlights

• Dissociation is strongly associated with negative appraisals of such experiences.
• Heightened affect sensitivity and low self-efficacy may influence dissociation.
• Dissociation is likely maintained by cognitive-behavioural threat-based responses.
• Dissociation likely leads to beliefs about being overwhelmed and ‘shutting off’.

The identification of plausible mechanisms of dissociation is an essential step in developing an evidence-based cognitive model that can be successfully translated into treatment. Numerous mechanisms have been proposed to underlie dissociation (e.g. Aksen et al., 2020; Dalenberg et al., 2012; Lynn et al., 2019). In this study, we use novel statistical approaches to identify from an array of mechanisms those that may be most closely tied to dissociation. Further, we focus on one type of dissociative experiences: those characterised by a felt sense of anomaly (FSA) (Černis et al., 2021a). Felt sense of anomaly is defined as a subjective sense of strangeness which may take the form of unreality, disconnection, automaticity, or unfamiliarity, and occurs within one or more domains such as perception, identity, the body, and cognitions (Černis et al., 2021a). Examples of FSA are “the world seems like it is fake” (unreality, external world), “my body feels like it's not under my control” (automaticity, body), and “I feel detached from my emotions” (disconnection, affect). It is increasingly recognised that the term dissociation comprises multiple distinct experiences (e.g. Holmes et al., 2005), and hence a focus on individual types – such as FSA-dissociation – may enable greater precision in understanding. This construct includes symptoms that may also be described in terms of detachment/compartmentalisation (Holmes et al., 2005) or depersonalisation and derealisation (Sierra and Berrios, 1997, 1998). This study forms part of a programme of work developing a theoretical understanding of FSA-dissociation that might lead to more efficacious treatment by informing the development of a targeted translational psychological intervention.

Network analyses estimate structural models of multivariate dependencies (Burger et al., 2020) and are therefore well-suited to exploring and visualising complex sets of inter-correlated variables (Robinaugh et al., 2019). We first examine inter-correlations with an undirected network, to robustly estimate the underlying structure of the data, before proceeding to Bayesian inference with directed acyclic graphs (DAGs) analysis, which can generate tentative hypotheses about probable directions of relationships. The use of network analysis methods in this study marks a point of difference from the existing literature, in which the majority of evidence consists of correlations between dissociation and a very small number of mechanisms of interest. Importantly, since network analysis methods make use of conditional dependence, the most influential variables are more easily identified in this approach, as mediation effects are incorporated into the network estimation. This complexity is untested in simple correlation analyses, potentially resulting in over-inflated estimates of the importance of mechanisms. Exploring multiple relevant variables simultaneously, whilst accounting for their inter-correlations, therefore advances the understanding gained in previous studies. Key variables could then form the focus of causal tests in future studies.

In this study following on from our previous work published in this journal (Černis, Evans, Ehlers & Freeman, 2021b) demonstrating the importance of dissociation across mental health conditions, we focus on cognitive variables repeatedly found to be associated with dissociation, or that are highly plausible mechanistically. Ten processes were identified: cognitive appraisals, responses to dissociation (such as ‘safety behaviours’), affect intolerance, alexithymia, attentional control, body vigilance, anxiety sensitivity, general self-efficacy, perseverative thinking, and beliefs regarding stress.

Factors associated with processing of emotion have been particularly implicated in dissociation. For example, anxiety sensitivity has been demonstrated to predict dissociative reactions to a hyperventilation procedure in participants with acute stress disorder (Nixon and Bryant, 2006), and alexithymia – the inability to recognise or name subjective experiences of affect – has robust correlations with dissociation (Evren et al., 2012). Anxiety sensitivity and alexithymia may provide contexts in which endogenous (internal) signals related to affect may be attended to and subsequently appraised as odd or unusual. The explicit avoidance or even fear of experiencing emotions, affect intolerance, has also been linked to dissociation. For example, negative attitudes towards emotion have been shown to predict clinical levels of depersonalisation (Ó Laoide et al., 2018). Negative emotional responses towards emotion (‘meta-emotion’; Mitmansgruber et al., 2009) may be one way in which such attitudes manifest and may also be important. Finally, it has been proposed that dissociation may be a natural ‘shutting off’ response to becoming overwhelmed by emotion (Young, 1988). This was found to be a feared outcome of experiencing heightened affect in people with psychosis and dissociative symptomatology (Černis et al., 2020). Therefore, beliefs about being overwhelmed were also included in the current study. Since the construct of self-efficacy (beliefs regarding one's capabilities) may be relevant to these beliefs – for example in estimating the likelihood of becoming overwhelmed - this was also included.

According to general cognitive-behavioural therapy (CBT) theory, cognitive appraisals of events, and actions taken to mitigate anticipated ill-effects (e.g. safety behaviours), are crucial to the maintenance of a psychological difficulty. Catastrophic cognitive appraisals of dissociation (for example, ‘I'm losing my mind’; Černis et al., 2020) may heighten the significance and subjective threat posed by the dissociative experience, and drive behavioural and cognitive responses such as avoidance. Such responses provide short-term relief but prevent longer-term learning or development of coping skills which could lead to a sustainable reduction in the feared experience. Therefore, cognitive appraisals and common responses to dissociation were included as potential mechanisms in this study.

Common maintenance processes implicated in CBT theory include rumination and hypervigilance. These have also been associated with dissociation. Freeman et al. (2013) demonstrated increased depersonalisation following a worry induction procedure, and Jaspers (1963; as cited in Hunter et al., 2003) suggests that ‘obsessive focus’ on a topic (e.g. the self) may produce sensations of unreality with respect to that domain. This implies that perseverative thinking at a sufficient intensity could itself induce FSA. Indeed, previous research has found that staring at an (external) object induces feelings of derealisation (e.g. Möllmann et al., 2020). Poor attentional control also correlates moderately with dissociation (Segal and Lynn, 1993), and may explain the persistence of ‘obsessive focus’. Therefore, perseverative thinking, body vigilance, and attentional control were also included in the current study.

As in the preceding study, network analyses were used to identify the processes most strongly connected to felt sense of anomaly (FSA) dissociative experiences.

## Methods

### Design

The design was an online cross-sectional self-report questionnaire study.

The majority of participants were recruited via Facebook advertisements. The survey landing page contained the participant information sheet and statements regarding informed consent. The participant information sheet stated that researchers were seeking participants “to complete questionnaires about different kinds of thoughts”, and that they need not have experienced dissociation to take part. Informed consent and assessment were both carried out online using Qualtrics (2019). Surveys were accessible on desktop and mobile web browsers.

### Participants

Inclusion criteria were deliberately broad to be able to recruit as diverse a participant group as possible: any adult (age 18 years or over), usually resident in the United Kingdom. To increase diversity and facilitate online participation, there were no exclusion criteria.

During the recruitment period (January 30th^,^ 2019 to February 25th^,^ 2019), 10520 responses were recorded by Qualtrics. Of these, 361 (3.43%) indicated consent but then left the survey without continuing on to the first page of measures. After removing participants who did not meet the inclusion criteria, had high levels of missing data (greater than 20% in any of the measures), or recorded an age more than two standard deviations above the mean age of the sample (i.e. 76 years or above), a sample of 6161 was obtained.

Of the 6161 participants, the majority were female (85.6%, n = 5274) and White (95.3%; n = 5872). The mean age of the sample was 45.6 years (SD = 14.7). See Table 1 for further detail. Additionally, 80.6% of the sample reported that they had experienced mental health difficulties (in answer to the question ‘*Have you ever experienced mental health difficulties?’* in the demographics section of the survey).

**Table 1:** Showing descriptive statistics for the participant group and each scale in the survey.

Demographic		N		% of group (n = 6161)	
Gender	*Male*	717		11.64	
	*Female*	5274		85.60	
	*Other*	128		2.08	
	*Prefer not to say*	42		0.68	
Ethnicity	*Asian (any background)*	43		0.70	
	*Black (any background)*	14		0.23	
	*Mixed or Multiple ethnicity*	137		2.22	
	*White (any background)*	5877		95.39	
	*Other*	7		0.11	
	*Prefer not to say*	83		1.35	
		**Mean**		**SD**	
Age		45.61		14.65	
**Scale**		**Group mean (SD)**	**Cronbach's alpha in this group**		**Scale min – max score**
Černis Felt Sense of Anomaly scale		43.86 (27.05)	0.97		0–140
Cognitive Appraisals of Dissociation		16.91 (11.86)	0.93		0–52
Responses to Dissociation		14.67 (4.13)	0.67		0–24
Affect Intolerance Scale		120.70 (30.68)	0.95		30–180
Anxiety Sensitivity Index		31.56 (16.81)	0.93		0–72
Attentional Control Scale		48.99 (9.89)	0.87		20–80
Body Vigilance Scale		19.15 (8.71)	0.93		0–40
General Self-Efficacy Scale		27.48 (6.07)	0.92		10–40
Meta-Emotion Scale *(subset of 11 items)		44.24 (11.79)	0.89		11–66*
Online Alexithymia Questionnaire *(subset of 11 items)		33.47 (8.48)	0.84		11–55*
Perseverative Thinking Questionnaire		35.41 (13.78)	0.97		0–60
Beliefs About Being Overwhelmed		19.01 (8.38)	0.90		0–32

### Measures

Cronbach's alphas for each scale are shown in Table 1. All scales demonstrated good or excellent internal consistency in this sample.

#### Affect intolerance scale (AIS; Stapinski, Abbott & Rapee, 2014)

The Affect Intolerance Scale comprises 30 items rated on a Likert scale from 1 “strongly disagree” to 6 “strongly agree” which ask about the respondents' ‘beliefs and responses across a broad range of negative feelings’. Items are rated ‘in general’ and form two factors: “threat expectancy” (e.g. *“Once I have negative feelings, I worry that they will get worse”*) and “avoid/suppress” (e.g. *“I should avoid negative feelings”*). Higher scores indicate greater intolerance of negative affect.

#### Anxiety sensitivity index (ASI; Deacon et al., 2003)

The Anxiety Sensitivity Index contains 18 items which ask about concerns people may experience as a result of anxiety: these may be physical *(“It scares me when my heart beats rapidly”*), cognitive *(“When I cannot keep my mind on a task, I worry that I might be going crazy”*), or social (*“It is important for me not to appear nervous”*). Items are rated from 0 “very little” to 4 “very much”. Higher scores indicate greater sensitivity to anxiety.

#### Attentional control scale (ATC; Derryberry and Reed, 2002)

The Attentional Control Scale contains 20 items and asks respondents to rate from 1 “almost never” to 4 “always” items such as *“My concentration is good even if there is music in the room around me”* and reverse items such as *“I have trouble carrying on two conversations at once”*. Items are rated ‘based on general experience’. Higher scores indicate greater attentional control.

#### Body vigilance scale (BVS; Schmidt, Lerew & Trakowski, 1995)

The Body Vigilance Scale measures sensitivity to physiological sensations such as faintness or vision changes. Three questions such as *“I am the kind of person who pays close attention to internal body sensations”* are rated from 0 “never” to 10 “extremely” for the past week, then a fourth item asks respondents to rate how much attention they have paid to a list of different sensations over the same timeframe. Higher scores indicate greater vigilance for bodily sensations.

#### Černis felt sense of anomaly scale (ČEFSA; Černis et al., 2021a)

The ČEFSA measures dissociative experiences which share a core experience of a felt sense of anomaly (FSA) using 35 items rated for the past two weeks (e.g. “*I feel like a stranger to myself”, “the world around me seems unreal”*). Items are rated on a Likert scale from 0 “never” to 4 “always”. Higher scores indicate higher levels of FSA-dissociation.

#### Cognitive appraisals of dissociation (CAD-P; Černis et al., 2020)

The CAD-P measures cognitive appraisals of dissociative experiences using 13 items rated for ‘when you are feeling strange, disconnected, unreal or “dissociated"’ (e.g. *“This might last forever”*). Items are rated on a Likert scale from 0 “never” to 4 “always”. Higher scores indicate more frequent occurrence of catastrophic appraisals in response to dissociative experiences.

#### General self-efficacy scale (GSE; Schwarzer and Jerusalem, 1995)

The General Self-Efficacy scale comprises ten items such as *“I can always manage to solve difficult problems if I try hard enough”* and *“I can usually handle whatever comes my way”*. Items are rated for ‘how true … they are of you in general’ on a four-point Likert scale from 1 “not at all true” to 4 “exactly true”. Higher scores indicate greater self-efficacy.

#### Beliefs about being overwhelmed (overwhelm)

As described above, beliefs about feeling overwhelmed were considered to be potentially important to include in the current study. However, no existing measure of this concept were found, and therefore the authors generated a new scale (Supplementary Material 1). Eight items encompass ideas about inability to cope (e.g. *“I can't cope with stress”*) and “shut off” (e.g. “*If there's too much to deal with, I just shut off*”). Items are rated from 0 “not at all like me” to 4 “very much like me”. Higher scores indicate greater endorsement of the belief that stress or emotion can lead to cognitive “overwhelm”, potentially resulting in mental “shut down”.

#### Meta-emotion scale (MES; Mitmansgruber et al., 2009)

The Meta-Emotion Scale measures emotional reactions to one's own emotions, and comprises six subscales: “anger”, “compassionate care”, “interest”, “contempt/shame” [sic], “tough control” and “suppression”. For this study, only negative meta-emotions were included (i.e. anger, contempt/shame, tough control and suppression).

The adapted version of the MES (Supplementary Material 1) used in this study consisted of 11 items measuring anger, contempt/shame and tough control in response to having emotions (e.g. *“I repeatedly get angry about my emotional reactions”*). Items rate respondents' answers in relation to ‘times of stress’. Items are rated from 1 “is not at all true for me” to 6 “is completely true for me”, with higher scores indicating stronger negative emotional responses to one's emotions.

#### Online Alexithymia Questionnaire (OAQ-G2; Thompson, 2007)

For this study, only three of the Online Alexithymia Questionnaire's seven factors were considered: “difficulty identifying feelings” (e.g. *“When asked which emotion I'm feeling, I frequently don't know the answer”*); “difficulty describing feelings” (e.g. *“I can describe my emotions with ease”*); and “externally-oriented thinking” (e.g. *“I prefer doing physical activities with friends rather than discussing each others' emotional experiences”*). These were chosen as they most closely relate to the conceptualization of alexithymia as difficulty identifying emotions.

Therefore, the adapted version of the OAQ used in this study consisted of 11 items rated from 1 “strongly agree” to 5 “strongly disagree” (with one reverse-coded item). No specific timeframe is indicated in the measure instructions. Higher scores indicate greater difficulty identifying, naming and acknowledging one's emotional state.

#### Perseverative thinking questionnaire (PTQ; Ehring et al., 2011)

The Perseverative Thinking Questionnaire is a 15-item measure of repetitive negative thinking. Items relate to how the respondent ‘typically’ thinks about negative experiences or problems (“*The same thoughts keep going through my mind again and again*”) and are rated from 0 “Never” to 4 “almost always”. Higher scores indicate higher levels of ruminative thinking.

#### Responses to dissociation (RTD)

This new scale (Supplementary Material 1) measures six behavioural responses to dissociation (e.g. *“I try to keep busy”* and *“I isolate myself from others*”). Higher scores on the RTD therefore indicate greater use of particular behaviours in response to FSA-dissociation. Items are rated from 0 “never” to 4 “always” for how the respondent typically responds to feeling dissociated.

### Statistical analysis

Analyses were conducted in R, version 3.6.1 (R Core Team, 2019). The ‘mice’ package (version 3.8.0; van Buuren and Groothuis-Oudshoorn, 2011) was used to carry out multiple imputation for missing data. Prior to network estimation, data were transformed to a normal distribution (using ‘gaussianize’ in the DAGtools package, v0.1.001l) before an undirected partial correlation network and a Bayesian inference with directed acyclic graphs (DAGs) network were estimated.

#### Undirected partial correlation network

The undirected (Gaussian Graphical model) network was estimated and visualised using ‘bootnet’ (v1.3) and ‘qgraph’ (Epskamp et al., 2012). Due to the large sample size, the ggmModSelect method was used to obtain optimum model fit. Non-parametric bootstrapping (5000 bootstraps) was used to assess the accuracy and stability of the estimated network (Supplementary Material 2). In the final graph, positive partial correlations are shown by a blue and negative correlations by a red line. The strength of the pairwise partial correlations between nodes is indicated in both cases by the weight of the edge.

#### Bayesian inference with directed acyclic graphs

The final causal graph was calculated by averaging the results of 50,000 sample DAGs, obtained by using the BiDAG package to run the partition Markov Chain Monte Carlo algorithm (Kuipers and Moffa, 2017; Kuipers et al., 2018) for 10 million iterations. The final graph shows only edges that were present in over 50% of the 50,000 sampled DAGs, and only those which showed a specific direction in over 90% of cases are directed (i.e. contain an arrowhead in the probable direction of effect). Causal effects (z-scores with credible intervals; CIs) were also calculated. A credible interval may be interpreted in a similar manner to a confidence interval, but it is calculated according to the probability distribution given the data.

## Results

Table 1 displays summary scores for each scale. The correlation matrix showing intercorrelations between all variables can be found in Supplementary Material 2.

### Undirected network

**Fig. 1:**
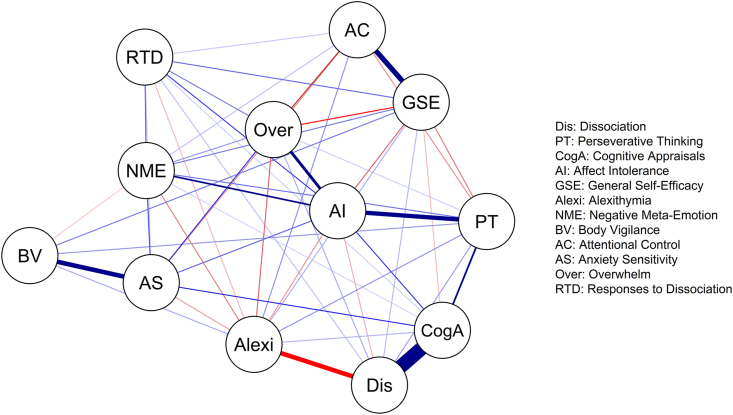
Undirected network graph showing relationships between dissociation and candidate maintenance mechanisms. (Red lines show negative relationships. Blue lines show positive relationships. Greater thickness and colour strength of edges indicates greater edge weight).

Fig. 1 shows the undirected network (see Supplementary Material 2 for full estimation details)*.* In summary, FSA-dissociation had direct relationships with most of the variables, but not with attentional control, anxiety sensitivity, body vigilance, or negative meta-emotion. Dissociation was negatively related to alexithymia, and most strongly connected to cognitive appraisals about dissociation (CAD). The edge between FSA-dissociation and CAD was statistically significantly stronger than any other edge in the network. Like dissociation, CAD also did not have a direct edge with attentional control or body vigilance, but did with negative meta-emotion and anxiety sensitivity. The expected direct edge between CAD and responses to dissociation (RTD) was found, with a weak edge.

Centrality estimates (Supplementary Material 2) indicated a highly inter-connected network, where affect intolerance was particularly important in terms of direct and indirect pathways. Cognitive appraisals also had a high betweenness centrality score, suggesting that it may act as a connector node between dissociation and other variables in the network. The stability of centrality estimates was high, and the accuracy of network estimation was good.

### Bayesian inference with directed acyclic graphs (DAGs)

**Fig. 2:**
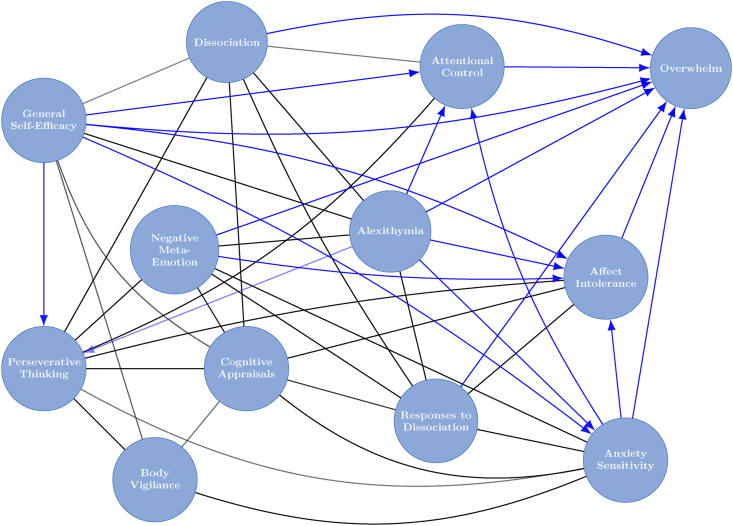
Mixed graph (i.e. with both directed and undirected edges) showing relationships between dissociation and psychological mechanisms that may plausibly be related to dissociation, as measured in the online survey. Undirected lines show direct relationships that were present in over 50.00% of the 50,000 sampled DAGs. Lines with arrowheads show the probable direction of influence if this was present in over 90.00% of the 50,000 sampled DAGs.

The results of the Bayesian inference with DAGs estimation is shown in Fig. 2. Only direct pathways (edges) which were present in over 50.00% of the 50,000 sampled DAGs are represented. Edges which were present *and* showed the same direction of influence in over 90.00% of the sampled DAGs are shown with an arrowhead indicating the direction of effect. Edges without arrowheads can therefore be interpreted as direct relationships that were present in over 50.00% of the sampled DAGs but have less clear direction of effect.

Focusing on the variable of FSA-dissociation within the network in order to identify potential causal factors, Table 2 summarises the directions of relationships between dissociation and all other variables.

**Table 2:** Summarising the average causal effects between dissociation and all other variables (Percentages to 2 d.p., causal effects & CIs to 2 s.f.).

Causal effects:	Pathway present (direct or indirect) %	Causal effect	90% CI	Direct edge present %	Direct causal effect	90% CI
Variable to dissociation (i.e. variable causing dissociation)						
General Self-Efficacy	57.96	−0.24	−0.38–−0.021	64.08	<0.01	–
Cognitive Appraisals	72.77	0.73	0.64–0.82	100	0.72	0.63–0.82
Perseverative Thinking	42.74	0.31	0.090–0.61	100	0.093	0.054–0.29
Body Vigilance	52.29	0.15	0.0099–0.21	3.32	<0.01	–
Anxiety Sensitivity	37.38	0.32	0.19–0.53	10.53	0.013	0.00–0.063
Negative Meta-Emotion	38.18	0.16	0.055–0.47	9.79	0.011	0.00–0.054
Alexithymia	39.12	−0.54	−0.61–−0.34	100	−0.27	−0.53–−0.22
Responses to Dissociation	43.32	0.20	0.072–0.44	99.95	0.11	0.034–0.43
Affect Intolerance	34.75	0.26	0.19–0.30	44.45	−0.021	−0.066–0.00
Attentional Control	11.43	−0.045	−0.091–−0.016	49.83	−0.014	−0.040–0.00
Overwhelm	1.46	0.27	0.09–0.59	100	0.12	0.071–0.15
**Dissociation to variable (i.e. dissociation causing variable)**						
General Self-Efficacy	27.45	−0.27	−0.38–−0.035	48.98	−0.080	−0.37–0.00
Cognitive Appraisals	27.23	0.74	0.55–0.80	100	0.70	0.54–0.80
Perseverative Thinking	52.26	0.31	0.088–0.62	100	0.096	0.064–0.12
Body Vigilance	30.21	0.16	−0.0062–0.22	10.52	0.016	0.00–0.21
Anxiety Sensitivity	62.14	0.25	0.061–0.56	5.98	<0.01	–
Negative Meta-Emotion	61.28	0.23	0.081–0.50	7.21	0.010	0.00–0.084
Alexithymia	60.88	−0.50	−0.60–−0.44	100	−0.46	−0.53–−0.42
Responses to Dissociation	56.68	0.24	0.11–0.44	100	0.18	0.088–0.42
Affect Intolerance	65.01	0.26	0.054–0.62	6.27	<0.01	–
Attentional Control	68.17	−0.20	−0.41–−0.055	70.40	−0.054	−0.11–0.00
Overwhelm	98.54	0.24	0.072–0.58	100	0.081	0.062–0.10
**Key:**						
*‘Pathway present’*	The proportion of sampled DAGs which found this pathway.					
*‘Causal effect’*	Average total causal effect when that pathway was present.					
*‘Direct edge present’*	The proportion of DAGs that found direct pathways of those where some pathway was found to be present.					
*‘Direct causal effect’*	Average total causal effect of the direct pathways.					
*CI*	Credible interval.					

The table can be interpreted as with the following example: from general self-efficacy (GSE) to dissociation, there was a pathway (direct or indirect) present in 57.96% of the 50,000 DAGs sampled – that is, GSE influences dissociation in 57.96% of the 50,000 sampled graphs. The average strength of the effect of GSE on dissociation within these 57.96% of graphs was −0.24 (with a 90% credible interval −0.38 to −0.021). In 64.08% of the 57.96% of DAGs sampled which showed a pathway from GSE to dissociation, a *direct* edge between GSE and dissociation was present, and this had an average strength of less than 0.01. In the opposite direction, from dissociation to GSE (bottom half of Table 2), there was a pathway present in 27.45% of the 50,000 sampled graphs, and the average strength of this effect was −0.27 (CI = −0.38 to −0.035). Again, 48.98% of the 27.45% of sampled DAGs contained a direct pathway (edge) from dissociation to GSE. The average strength of this direct pathway was −0.080 (CI = −0.37-0.00). Therefore, for the relationship between GSE and dissociation, there is a non-significant indication (i.e. found in more than 50% but less than 90% of sampled DAGs) that GSE has a small negative influence on dissociation. The direction of influence whereby dissociation affects GSE was found in fewer than 50% of sampled DAGs, indicating a low probability of this direction of effect.

The majority of the effect of GSE on FSA-dissociation is via the direct pathway but had an effect not significantly different from 0. This indicates the importance of the indirect pathways. Within the indirect pathways, a pathway from GSE to dissociation via cognitive appraisals of dissociation (CAD) was found in 83.39%, and a pathway via perseverative thinking was found in 65.63% of the 57.96% of DAGs with a GSE-dissociation pathway. Pathways via affect intolerance, anxiety sensitivity, and negative meta-emotion were also present in over 50% of sampled DAGs showing a pathway between GSE and dissociation.

Only GSE, body vigilance, and CAD were found to influence FSA-dissociation in over 50% of sampled DAGs (none reach the threshold of this direction being found in over 90% of the graphs). Consistent with the results of the undirected network, the effect sizes of the relationships between dissociation and CAD were the highest in the network (moderate effect size). By contrast, nearly all other variables were found to be more likely to be influenced by dissociation, albeit with only the effect on ‘overwhelm’ reaching the 90% threshold for drawing inferences about direction of effect.

Since CAD and dissociation are highly (directly) connected, and CAD may be acting as a connector node between other variables and dissociation, the directions of relationships between CAD and other variables were also calculated (Table 3).

**Table 3:** Summarising the average causal effects between cognitive appraisals about dissociation (CAD) and all other variables. (Percentages to 2 d.p., causal effects & CIs to 2 s.f.).

Causal effects:	Pathway present (direct or indirect) %	Causal effect	90% CI	Direct edge present %	Direct causal effect	90% CI
Variable to CAD (i.e. variable causing CAD)						
Dissociation	27.23	0.74	0.55–0.80	100	0.70	0.54–0.80
General Self-Efficacy	60.36	−0.31	−0.44–−0.15	45.68	−0.12	−0.44–0.00
Perseverative Thinking	40.30	0.38	0.32–0.69	100	0.35	0.30–0.58
Body Vigilance	51.93	0.19	0.025–0.26	34.01	0.064	0.00–0.25
Anxiety Sensitivity	37.98	0.44	0.26–0.62	100	0.21	0.18–0.29
Negative Meta-Emotion	36.70	0.22	0.12–0.52	75.94	0.042	0.00–0.10
Alexithymia	43.22	−0.41	−0.53–−0.042	80.72	−0.10	−0.15–0.00
Responses to Dissociation	38.04	0.23	0.074–0.46	97.06	0.15	0.054–0.46
Affect Intolerance	34.55	0.37	0.33–0.41	100	0.21	0.17–0.23
Attentional Control	6.69	−0.058	−0.17–−0.0099	1.19	–	–
Overwhelm	1.44	0.25	0.052–0.63	6.94	0.017	0.00–0.095
**CAD to variable (i.e. CAD causing variable)**						
Dissociation	72.77	0.73	0.64–0.82	100	0.72	0.63–0.82
General Self-Efficacy	39.64	−0.42	−0.47–−0.21	99.95	−0.40	−0.47–−0.20
Perseverative Thinking	59.70	0.61	0.47–0.72	100	0.31	0.25–0.58
Body Vigilance	46.57	0.25	0.15–0.30	86.92	0.23	0.00–0.30
Anxiety Sensitivity	62.02	0.54	0.39–0.66	100	0.36	0.25–0.40
Negative Meta-Emotion	63.30	0.44	0.26–0.57	98.83	0.22	0.088–0.31
Alexithymia	56.66	−0.36	−0.55–−0.044	2.82	<0.01	–
Responses to Dissociation	61.96	0.31	0.075–0.47	66.69	0.21	0.00–0.47
Affect Intolerance	65.45	0.57	0.30–0.72	100	0.26	0.16–0.30
Attentional Control	67.95	−0.27	−0.44–−0.040	1.53	<0.001	–
Overwhelm	98.48	0.31	0.048–0.63	1.34	<0.001	–
**Key:**						
*‘Pathway present’*	The proportion of sampled DAGs which found this pathway.					
*‘Causal effect’*	Average total causal effect when that pathway was present.					
*‘Direct edge present’*	The proportion of DAGs that found direct pathways of those where some pathway was found to be present.					
*‘Direct causal effect’*	Average total causal effect of the direct pathways.					
*CI*	Credible interval.					

The results for CAD are similar to those for dissociation in that only two variables – GSE and body vigilance – were found to influence CAD in over 50% of sampled DAGs; and neither reach the 90% threshold. The effect size for body vigilance is less than 0.2 and small to medium (-0.31) for GSE. Unlike the result for dissociation, the minority of the effect of GSE on CAD is via the direct pathway (45.68% of the 60.36% of DAGs that found a pathway). Similar to the result for dissociation, the direct pathway itself has very small (less than 0.2) causal effect.

Again, the results indicate that for all other variables, the opposite direction of effect is more likely (that CAD is causal). As with dissociation, only the effect on ‘overwhelm’ reached the 90% threshold for drawing inferences about direction of effect, and this was almost exclusively via indirect pathways.

As expected, a direct pathway between CAD and responses to dissociation (RTD) was found. The results indicate that it is more probable (although not reaching the 90% threshold) that CAD is a causal influence on RTD, with the direct pathway alone constituting a small causal effect (0.21).

## Discussion

In this study, state-of-the-art network analyses were used to begin to identify which psychological variables may be most closely connected to the occurrence of the ‘felt sense of anomaly’ (FSA) form of dissociation. The highly interconnected nature of both network models indicates that all of the variables included were relevant in some way to the broader understanding of felt sense of anomaly dissociative experiences. None of the factors were disconnected or remotely-connected. In both network analyses, the variable with the strongest relationship to FSA-dissociation was that of cognitive appraisals of dissociation, which is perhaps less surprising since this measure specifies that the items relate to dissociative experiences. Dissociation was also found in both analyses to have direct relationships with responses to dissociation, perseverative thinking, low general self-efficacy, lower levels of alexithymia, and beliefs about being overwhelmed. Of these, only the result for alexithymia is inconsistent with the literature, in that the direction of the relationship is opposite to that expected. The second network analysis (using Bayesian inference with DAGs) suggested that dissociation was most likely to cause beliefs about being overwhelmed, whilst the other relationships were ambiguous in direction, which may well indicate bidirectional effects. Psychological processes and factors related to attention appeared to be less likely to act as mechanisms in the occurrence of dissociation than those related to processing of affect. Overall, our view is that the results indicate that FSA-dissociation may be maintained by rumination on catastrophic thoughts about dissociative experiences in the context of subjectively low coping ability and subjectively high emotion sensitivity and hypervigilance.

Both networks found direct relationships between dissociation, cognitive appraisals, perseverative thinking, and responses to dissociation. The directions of these relationships were ambiguous, which may indicate reciprocal influence that cannot be modelled by the DAGs analysis due to its specification of acyclic relationships. This interpretation would be consistent with the general framework of cognitive theory for clinical problems which states that recurrent catastrophic appraisals and counterproductive behavioural responses reinforce the psychological problem from which they arise.

The results of this study also extend current understanding beyond the general cognitive model by implicating self-efficacy as also potentially important in dissociation. In particular, the negative edge-weight in the first network model, and negative causal effect in the second, indicates that it is specifically low self-efficacy that may provide the context for dissociation to thrive. Low self-efficacy was found to influence dissociation in over half of the sampled DAGs, with nearly all of its small causal effect acting via indirect pathways through cognitive appraisals, perseverative thinking, and variables relating to affect tolerance. This suggests that people who judge themselves to be less able to cope in difficult situations may experience greater dissociation, and that this could be due to an increased likelihood of having negative cognitive appraisals about dissociative experiences, a tendency to ruminate, and difficulties tolerating emotion. Certainly, this is consistent with participants’ descriptions of themselves and their experiences in qualitative interviews (Černis et al., 2020).

Alexithymia was also found to have a strong relationship with dissociation in both networks. It is a somewhat surprising result that the correlations were negative, considering many previous findings of moderate positive correlations between alexithymia and dissociation (e.g. Evren et al., 2012). This result suggests that participants with dissociation self-report as being very capable in identifying their emotional experiences, and perhaps reflects a heightened attunement or sensitivity to affect. Such an explanation is consistent with the findings of Černis et al. (2020), and may constitute hypervigilance to threat in the context of the hypothesised affect intolerance. Alternatively, the contrasting result may be due to this study adopting a specific conceptualization of dissociation as experiences involving a ‘felt sense of anomaly’. For example, Panayiotou et al. (2015) found a positive association between alexithymia and psychosomatic symptoms, which may be viewed as a form of dissociation (Nijenhuis, 2001).

**Fig. 3:**
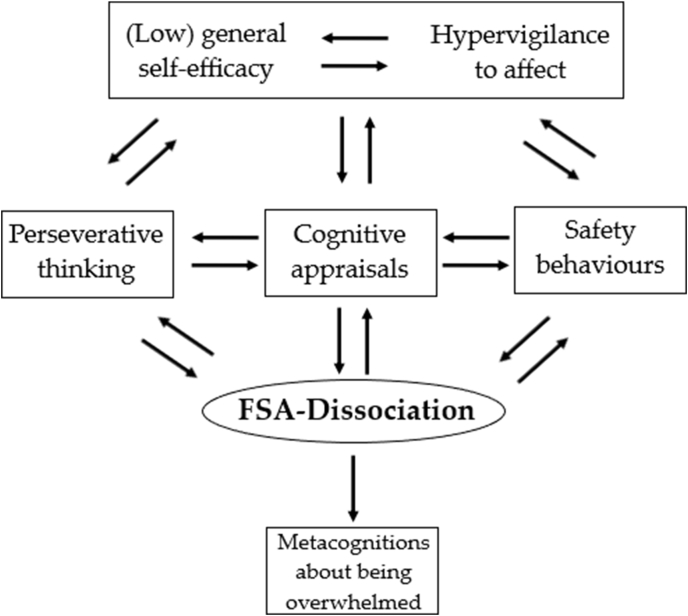
A diagram summarising the authors' interpretation of the study results.

Overall, therefore, the results of this study potentially indicate that factors relating to the cognitive processing of affect are important in FSA-dissociation as well as the processes indicated by the general clinical cognitive model (Fig. 3). The relationships with alexithymia (subjective emotion recognition ability), and general self-efficacy (possibly mediated by anxiety sensitivity, negative meta-emotion, and affect intolerance) in this study suggest that vulnerability to FSA-dissociation may be conferred in situations where heightened affect is detected and met with a negative response because the person believes themselves to be unlikely to cope. These trait-level variables may be reinforced through the effects of the more state-dependent variables of the general cognitive model: rumination upon upsetting cognitive appraisals causing distress and reinforcing the person's negative expectations regarding heightened affect. Additionally, appraisals and the subjective experience of dissociation themselves may be taken as evidence of an inability to cope, further reinforcing these beliefs. The use of safety behaviours (responses to dissociation) may also be theorised to have a similar reinforcing effect. Reciprocally, confidence in one's coping ability and hypervigilance to affect – at a trait level – are likely to influence the processes taking place in the general cognitive model: influencing catastrophisation, preoccupation, and reliance on safety behaviours to mitigate feared outcomes. The interpretation of these series of events appears to be likely to result in explicit beliefs that heightened stress and affect are overwhelming and cause mental ‘shut down’. Indeed, the description ‘overwhelming’ may be clinically useful in the context of FSA-dissociation, as this is implicated as an ultimate endpoint of the processes explored here.

The results have informed a subsequent clinical study of dissociation in a large cohort of patients diagnosed with non-affective psychosis (Černis et al., 2022). The findings of this new clinical study are consistent with the results reported with the non-clinical study. In the clinical group of nine hundred patients with non-affective psychosis, the direct relationships between dissociation and alexithymia (subjective emotion recognition ability), cognitive appraisals, safety behaviours, and perseverative thinking were replicated. The results are consistent with the ‘general cognitive model’ process, and with the hypothesis that threat-based processing of affect is important in FSA-dissociative experiences. Thus, the results of the two studies give a strong indication that variables relating to affect intolerance, affect sensitivity, and general self-efficacy, are bound together in relation to dissociation. This interconnectivity is robust enough that it is detected in both general population and non-affective psychosis groups. However, the exact configurations of these relationships remain to be tested by experimental methods that can determine causality.

There are limitations to the current study. As a result of recruiting via social media, the sample is biased in who participated – particularly with respect to gender and ethnicity, and in terms of self-selection bias – which limits the generalisability of the results. The cross-sectional design limits the strength of any causal conclusions. Reliance upon self-report assessment of psychological processes will likely lead to imprecision in measurement. Additionally, the results of DAGs analyses require tentative interpretation, and rely upon a number of assumptions which are unlikely to be fully upheld in psychological research. In particular, the assumption of causal sufficiency (that all relevant variables are present in the network) is unlikely to have been satisfied in the current study. This study was not exhaustive in terms of the psychological processes included in the analysis, and since it is unclear which factors may be the most relevant to dissociation, it is likely that important variables were omitted. Nonetheless, we believe this study provides a multi-factorial exploration of psychological factors relevant to a subgroup of dissociative experiences. This represents a necessary step towards an evidence-based cognitive model of a tightly-defined category of dissociative symptoms – a field of research which ‘has never suffered from clarity’ (Dell, 2009). This study has generated testable hypotheses to aid in this objective by identifying plausible maintenance mechanisms of dissociation. Future research is required both to identify further factors, and to more robustly test the factors implicated here using experimental or interventionist studies.

## Data availability statement

The authors do not have participant consent to share the data collected in this study.

## Funding statement

The work was supported by the 10.13039/100010269Wellcome Trust via a Clinical Doctoral Fellowship to EČ (grant number 102176/B/13/Z). DF was supported during this work by an 10.13039/501100000272NIHR Research Professorship (NIHR-RP-2014-05-003) and is an 10.13039/501100000272NIHR Senior Investigator. AE is funded by the 10.13039/100010269Wellcome Trust (200796) and supported by the 10.13039/501100013373Oxford Health NIHR Biomedical Research Centre and a 10.13039/501100000272NIHR Senior Investigator Award. The views expressed are those of the authors and not necessarily those of the National Health Service, NIHR, or Department of Health.

## Author: individual contributions (CRediT categories)

Emma Černis Conceptualization, Methodology, Software, Formal analysis, Investigation, Data curation, Visualization, Writing - original draft, Project administration, Funding acquisition.

Anke Ehlers Conceptualization, Methodology, Writing - review & editing, Supervision.

Daniel Freeman Conceptualization, Methodology, Writing - review & editing, Supervision.

## Declaration of competing interest

None.
