# Supplementary material for: Psychological mechanisms connected to dissociation: Generating hypotheses using network analyses
Source: J Psychiatr Res. 2022 Apr;148:165–73. doi: 10.1016/j.jpsychires.2022.01.049 (PMC8968218; doi:10.1016/j.jpsychires.2022.01.049)

Supplementary Material 1 – Author generated and adapted scales

The Responses to Dissociation scale (Author generated)

In a qualitative review (Černis, Freeman & Ehlers, 2020), participants with psychosis described a number of behaviours they carried out in response to experiencing dissociation. Twenty items were generated using transcripts from this study. Iterative exploratory factor analysis (EFA) with half of the full sample (n=4143) resulted in a two-factor scale comprising six items (below). Factors in the final scale were “engage” (e.g. *“I try to keep busy”*) and “avoid” (e.g. *“I stay away from other people”*). Confirmatory factor analysis (CFA) in the second half of the full sample (n=4144) demonstrated a good fit for this model (χ^2^=165.768, df=8, CFI=0.972, TLI=0.948, RMSEA=0.079, SRMR=0.049) and good internal reliability (Cronbach’s alpha = 0.66).

Please rate how often you do the following when you are feeling strange, disconnected, unreal or "dissociated".

*Please note that this should NOT be whilst under the influence of drugs, alcohol or legal highs.*

|  |  | Never | Rarely | Sometimes | Often | Always |
| --- | --- | --- | --- | --- | --- | --- |
| 1 | I try to keep busy | 0 | 1 | 2 | 3 | 4 |
| 2 | I isolate myself from others | 0 | 1 | 2 | 3 | 4 |
| 3 | I try to focus on something else | 0 | 1 | 2 | 3 | 4 |
| 4 | I sit around not doing anything much | 0 | 1 | 2 | 3 | 4 |
| 5 | I keep my mind busy | 0 | 1 | 2 | 3 | 4 |
| 6 | I stay away from other people | 0 | 1 | 2 | 3 | 4 |

Factor 1 (‘engage in something else’): items 1, 3, 5.

Factor 2 (‘disengage and avoid’): items 2, 4, 6.

**Final CFA model for the Responses to Dissociation scale, with factor loadings**

0.636

1.072

1.057

0.866

0.693

0.920

Meta-Emotion Scale (Mitmansgruber, Beck, Höfer & Schüẞler, 2009) (Adapted)

The Meta-Emotion Scale measures emotional reactions to one’s own emotions, and comprises of six subscales: “anger”, “compassionate care”, “interest”, “contempt/shame” [sic], “tough control” and “suppression”. For this study, only negative meta-emotions were considered (i.e. anger, contempt/shame, tough control and suppression).

To ensure the resulting abridged measure was psychometrically valid, EFA was carried out with the first half of the full “understanding dissociative experiences” sample (n=4143), and the resulting factor structure assessed using CFA in the second half (n=4144). This analysis found that the three original factors of anger, contempt/shame and tough control remained. The suppression factor was not replicated in this analysis: one of the original factor’s two items was discarded for poor fit (“I cannot come to grips with strong emotions”), and the other (“I fight strongly against my emotions”) loaded instead onto the new tough control factor. Additionally, one other item (“I often think my emotional reaction is wrong”) was also discarded for poor fit. The resulting three-factor, 11-item adaptation of the measure was a good fit to the data in the second half of the sample (χ2=372.289, df=41, CFI=0.972, TLI=0.962, RMSEA=0.050, SRMR=0.028) and had high internal reliability (Cronbach’s alpha=0.89).

Items included in the adapted scale:

- I repeatedly get angry about my emotional reactions
- I downright cajole myself when I experience major emotional burden
- Sometimes I could get really mad at myself about the way I react emotionally
- I repeatedly force myself to pull myself together
- When I see my emotions as inappropriate, I get very strict with myself
- I fight strongly against my emotions
- Repeatedly, there are situations when I excoriate (criticise) myself
- When I feel guilty because I have made a mistake, I am quite unforgiving with myself
- When talking to myself in my thoughts, I am often harsh with myself
- I cannot forgive myself for a long time when I have done something wrong
- I place high demands on myself to gain control over my feelings

Online Alexithymia Questionnaire (Thompson, 2007) (Adapted)

Four of the original OAQ’s factors (“vicarious interpretation of feelings”, “restricted imaginative processes”, “problematic interpersonal relationships”, and “sexual difficulties and disinterest”) were felt to overlap conceptually with other presentations, such as autistic spectrum disorder (e.g. *“When other people are hurt or upset, I have difficulty imagining what they are feeling”*), and were omitted. Therefore only three of the original seven factors were considered: “difficulty identifying feelings”; “difficulty describing feelings”; and “externally-oriented thinking”.

To ensure that the abridged OAQ was psychometrically valid, EFA was carried out with the first half of the full *“understanding dissociative experiences”* sample (n=4143), and the resulting factor structure assessed using CFA in the second half (n=4144). The resulting three-factor structure, comprising 11-items (i.e. discarding six items for poor fit), had good fit to the data in the second half of the sample (χ^2^=433.982, df=41, CFI=0.968, TLI=0.957, RMSEA=0.053, SRMR=0.034) and high internal reliability (Cronbach’s alpha=0.84). The three factors in the adapted version corresponded to the original factors of “difficulty identifying feelings”, “difficulty describing feelings”, and “externally-oriented thinking”.

Items included in the adapted scale:

- When asked which emotion I’m feeling, I frequently don’t know the answer
- I sometimes experience confusing sensations in my body
- I prefer doing physical activities with friends rather than discussing each other’s emotional experiences
- I’m unsure of which words to use when describing my feelings
- When involved in difficult or turbulent relationships, I sometimes develop confusing physical symptoms
- I don’t like conversations in which more time is spent discussing emotional matters than daily activities because it detracts from my enjoyment
- I can describe my emotions with ease [Reverse scored]
- When I am upset I find it difficult to identify the feelings causing it
- I have puzzling physical sensations that even friends / acquaintances / others don’t understand
- When helping others I prefer to assist with physical tasks rather than offering counsel about their feelings
- Describing the feelings I have about other people is often difficult

The Beliefs About Being Overwhelmed scale (Author generated)

Twelve items were generated using transcripts from the study described by Černis, Freeman and Ehlers (2020) in order to develop a measure for the purposes of the current study. EFA with half of the full *“understanding dissociative experiences”* sample (n=4143) resulted in a two-factor scale comprising eight items (below). Factors in the final scale were “can’t cope” (e.g. *“I can’t cope with stress”*) and “shut off” (e.g. “*If there’s too much to deal with, I just shut off*”). CFA in the second half of the full sample (n=4144) demonstrated a good fit for this model (χ^2^=360.358, df=19, CFI=0.966, TLI=0.951, RMSEA=0.075, SRMR=0.031) and excellent internal reliability (Cronbach’s alpha=0.93). Items are rated from 0 “not at all like me” to 4 “very much like me”.

Below are some statements about stress and emotion. Please rate how closely these statements match your own beliefs:

|  |  | Not at all like me |  |  |  | Very much like me |
| --- | --- | --- | --- | --- | --- | --- |
| 1 | I have to be careful not to get overwhelmed | 0 | 1 | 2 | 3 | 4 |
| 2 | I can’t cope with stress | 0 | 1 | 2 | 3 | 4 |
| 3 | I’ve never been able to deal with emotions | 0 | 1 | 2 | 3 | 4 |
| 4 | I can't deal with anxiety | 0 | 1 | 2 | 3 | 4 |
| 5 | I just can't cope with feeling low or depressed | 0 | 1 | 2 | 3 | 4 |
| 6 | I just "cut off" when I get overwhelmed | 0 | 1 | 2 | 3 | 4 |
| 7 | Above a certain level of emotion, my mind shuts down | 0 | 1 | 2 | 3 | 4 |
| 8 | If there's too much to deal with, I just shut off | 0 | 1 | 2 | 3 | 4 |

Factor 1 (‘can’t cope’): items 1-5.

Factor 2 (‘shut off’): items 6, 7, 8.

**Final CFA model for the Beliefs About Being Overwhelmed scale, with factor loadings**

1.259

1.249

1.095

0.927

1.105

0.950

0.949

1.070

Supplementary Material 2 – Network estimation

Correlation matrix:

|  | Dis | PT | CogA | AI | GSE | Alexi | NME | BV | AC | AS | Over | SB |
| --- | --- | --- | --- | --- | --- | --- | --- | --- | --- | --- | --- | --- |
| Dis | - | 0.61 | 0.81 | 0.60 | -0.38 | -0.60 | 0.49 | 0.21 | -0.40 | 0.56 | 0.59 | 0.42 |
| PT | 0.61 | - | 0.70 | 0.75 | -0.48 | -0.46 | 0.57 | 0.24 | -0.48 | 0.59 | 0.64 | 0.41 |
| CogA | 0.81 | 0.70 | - | 0.69 | -0.44 | -0.52 | 0.54 | 0.24 | -0.43 | 0.65 | 0.62 | 0.44 |
| AI | 0.60 | 0.75 | 0.69 | - | -0.51 | -0.55 | 0.66 | 0.22 | -0.48 | 0.65 | 0.75 | 0.53 |
| GSE | -0.38 | -0.48 | -0.44 | -0.51 | - | 0.39 | -0.30 | -0.05 | 0.56 | -0.41 | -0.55 | -0.20 |
| Alexi | -0.60 | -0.46 | -0.52 | -0.55 | 0.39 | - | -0.47 | -0.10 | 0.42 | -0.49 | -0.57 | -0.38 |
| NME | 0.49 | 0.57 | 0.54 | 0.66 | -0.30 | -0.47 | - | 0.18 | -0.32 | 0.55 | 0.57 | 0.46 |
| BV | 0.21 | 0.24 | 0.24 | 0.22 | -0.05 | -0.10 | 0.18 | - | -0.12 | 0.37 | 0.21 | 0.14 |
| AC | -0.40 | -0.48 | -0.43 | -0.48 | 0.56 | 0.42 | -0.32 | -0.12 | - | -0.45 | -0.56 | -0.24 |
| AS | 0.56 | 0.59 | 0.65 | 0.65 | -0.41 | -0.49 | 0.55 | 0.37 | -0.45 | - | 0.64 | 0.43 |
| Over | 0.59 | 0.64 | 0.62 | 0.75 | -0.55 | -0.57 | 0.57 | 0.21 | -0.56 | 0.64 | - | 0.48 |
| SB | 0.42 | 0.41 | 0.44 | 0.53 | -0.20 | -0.38 | 0.46   \| *Dis* \| Dissociation \| \| --- \| --- \| \| *PT* \| Perseverative Thinking \| \| *CogA* \| Cognitive Appraisals \| \| *AI* \| Affect Intolerance \| \| *GSE* \| General Self Efficacy \| \| *Alexi* \| Alexithymia \| \| *NME* \| Negative Meta-Emotion \| \| *BV* \| Body Vigilance \| \| *AC* \| Attentional Control \| \| *AS* \| Anxiety Sensitivity \| \| *Over* \| Metacognitions about being Overwhelmed \| \| *SB* \| Safety Behaviours \| | 0.14 | -0.24 | 0.43 | 0.48 | - |

**Key:**

## Undirected network

Bootstrapped confidence intervals (CIs) for each edge were calculated to assess the accuracy of the connections in the estimated undirected network. These were calculated using non-parametric bootstrapping (5000 bootstraps) using bootnet (v1.3) and are shown in Figure 1. Overall, the network appears to be estimated with good accuracy, despite overlap of CIs between edges in the network. The edge between dissociation and cognitive appraisals was very strong, and did not overlap with the CIs for any other edge.


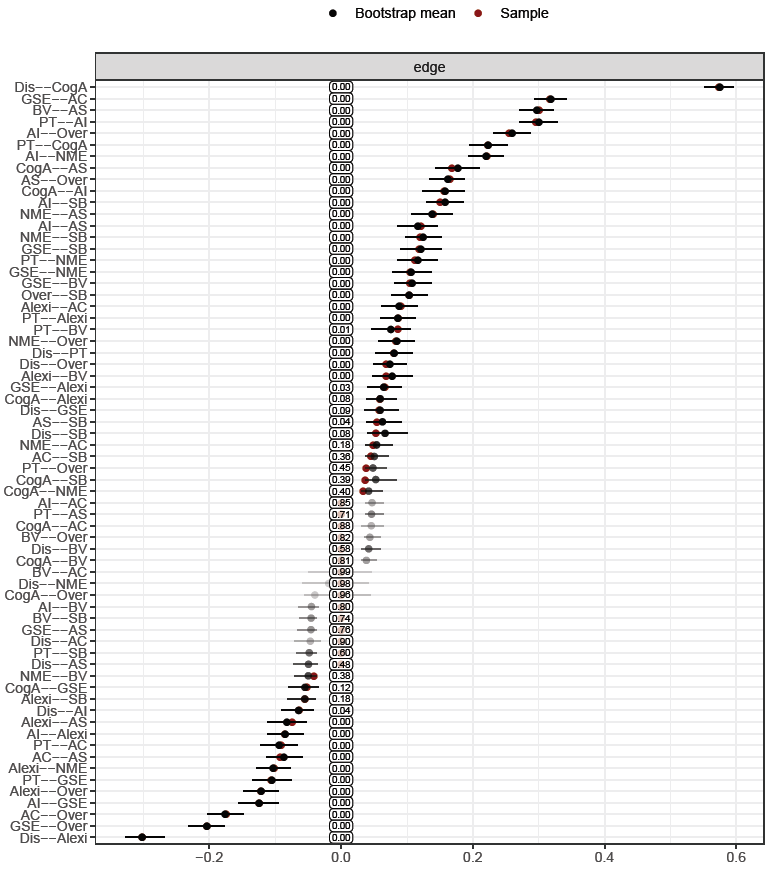
***Figure 1.*** Showing sample edge-weights and bootstrapped confidence intervals of edge-weights obtained via non-parametric bootstrapping (5000 bootstraps) for all edges in the undirected network

Table 1 shows the edge-weights and their bootstrapped confidence intervals for edges between dissociation and all other variables. This again indicates that the edge between dissociation and cognitive appraisals is statistically significantly stronger than any other edge involving dissociation. The next strongest edge is the negative relationship between dissociation and alexithymia, which is also statistically significantly stronger than other edges involving dissociation (apart from that between dissociation and cognitive appraisals).

| ***Table 1.*** Edge-weights and their bootstrapped confidence intervals for edges between dissociation and each other variable (3 s.f.) | | |
| --- | --- | --- |
| **Variable** | **Edge-weight** | **C.I.** |
| Perseverative Thinking | 0.0803 | 0.0511 – 0.109 |
| Cognitive Appraisals | 0.573 | 0.551 – 0.595 |
| Affect Intolerance | -0.0636 | -0.0992 - -0.0280 |
| General Self-Efficacy | -0.0576 | -0.0155 – 0.100 |
| Alexithymia | -0.302 | -0.330 – -0.273 |
| Negative Meta-Emotion | 0 (no edge) | -0.0127 – 0.0127 |
| Body Vigilance | 0 (no edge) | -0.0426 – 0.0426 |
| Attentional Control | 0 (no edge) | -0.0293 – 0.0293 |
| Anxiety Sensitivity | 0 (no edge) | -0.0514 – 0.0514 |
| Overwhelm | 0.0688 | 0.0423 – 0.0952 |
| Safety Behaviours | 0.0527 | 0.00337 – 0.102 |

The results of the edge-weight difference tests for all edges are shown in Figure 2.

***Figure 2.*** Differences between edge-weights: statistically significant differences indicated by a black square, non-significant differences by a grey square

| ***Key:*** | |
| --- | --- |
| *AC* | *Attentional control* |
| *AI* | *Affect intolerance* |
| *Alexi* | *Alexithymia* |
| *AS* | *Anxiety sensitivity* |
| *BV* | *Body vigilance* |
| *CogA* | *Cognitive appraisals* |
| *Dis* | *Dissociation* |
| *GSE* | *General self-efficacy* |
| *NME* | *Negative meta-emotion* |
| *Over* | *Overwhelm* |
| *PT* | *Perseverative thinking* |
| *SB* | *Safety behaviours* |


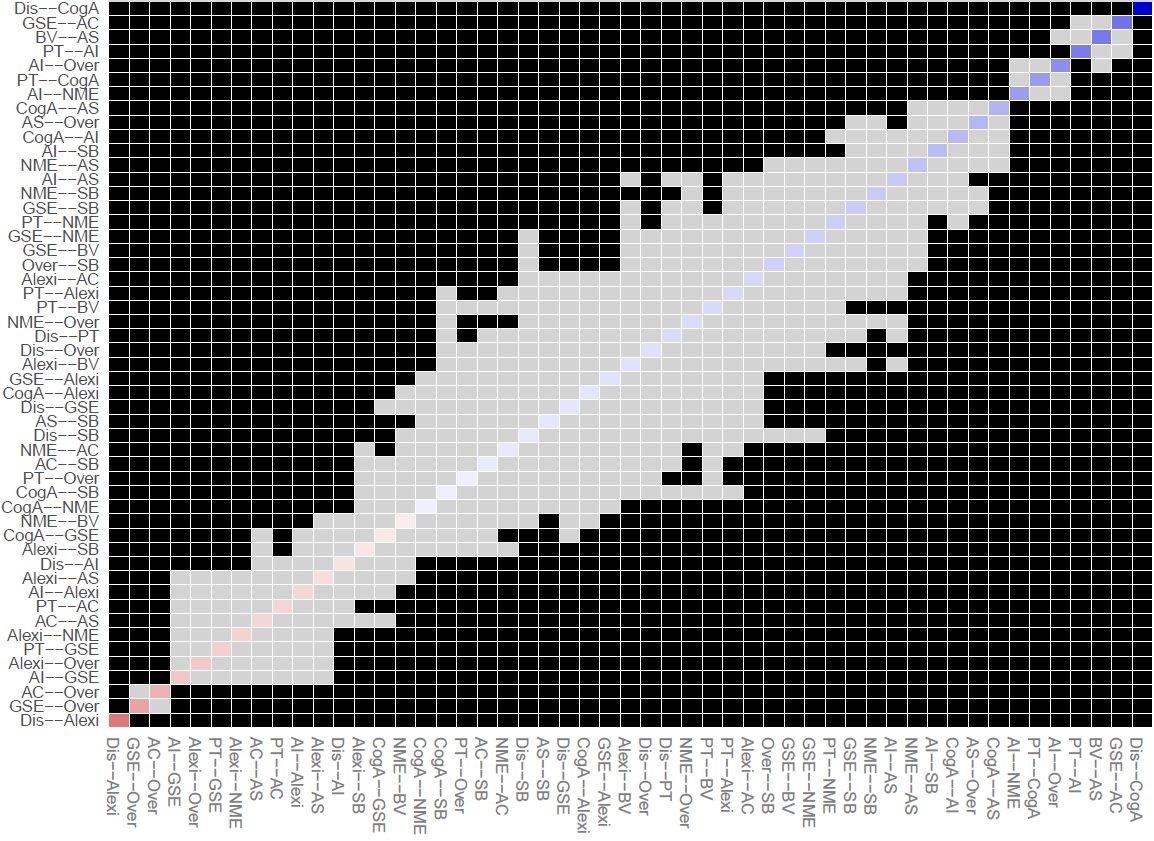


Centrality estimates (strength, closeness, and betweenness) were calculated for all variables. The results are shown in Figure 3 and Table 2. The results of difference tests for all centrality estimates for all variables are shown in Figure 4.

***Figure 3.*** Showing centrality scores for all variables in the network


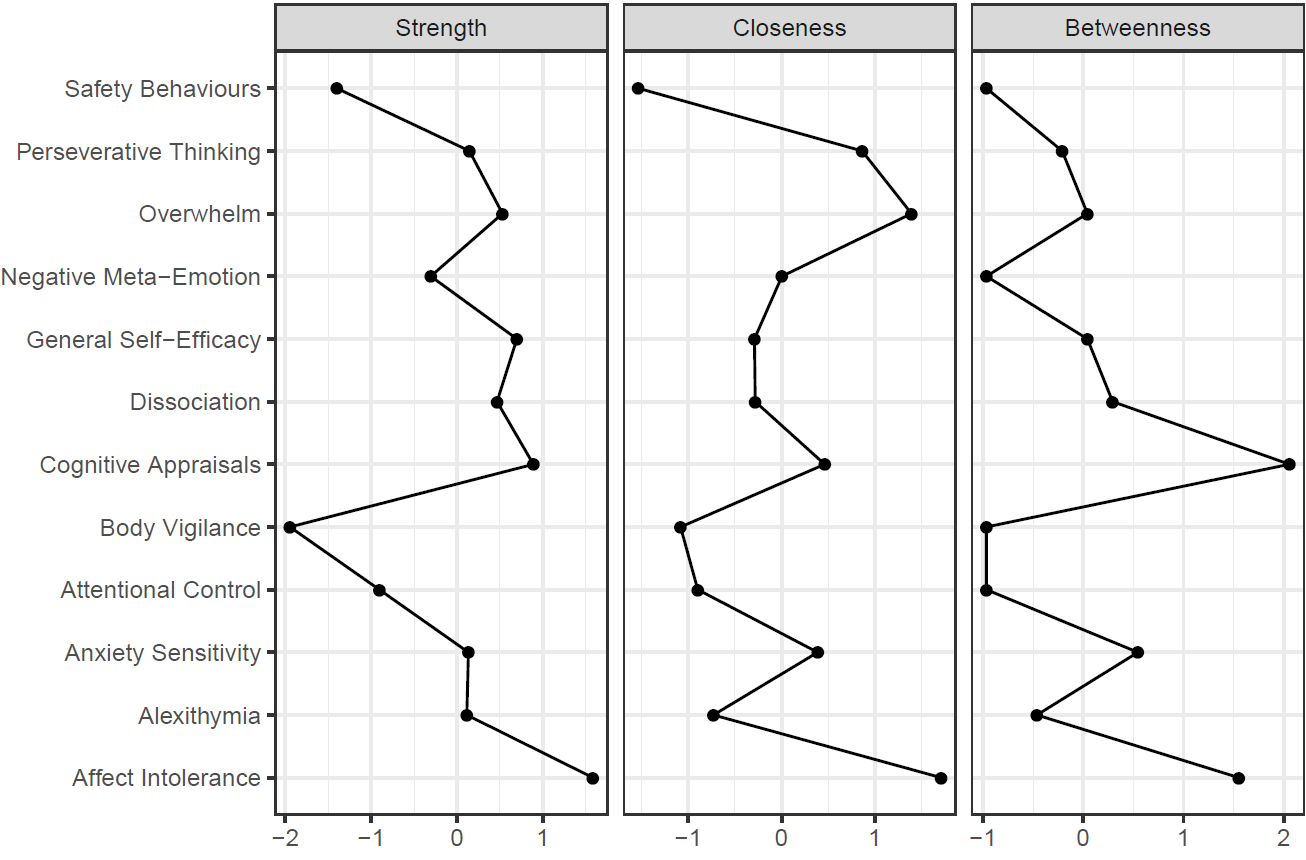


| ***Table 2.*** Centrality measures for all variables. (Degree centrality and closeness to 3 s.f.) | | | |  |
| --- | --- | --- | --- | --- |
| **Variable** | **Strength (Degree)** | **Closeness** | **Betweenness** | |
| Dissociation | 1.20 | 0.00930 | 10 | |
| Perseverative Thinking | 1.12 | 0.0110 | 6 | |
| Cognitive Appraisals | 1.30 | 0.0104 | 24 | |
| Affect Intolerance | 1.47 | 0.0122 | 20 | |
| General Self-Efficacy | 1.26 | 0.00929 | 8 | |
| Alexithymia | 1.11 | 0.00865 | 4 | |
| Negative Meta-Emotion | 1.01 | 0.00971 | 0 | |
| Body Vigilance | 0.601 | 0.00814 | 0 | |
| Attentional Control | 0.859 | 0.00841 | 0 | |
| Anxiety Sensitivity | 1.12 | 0.0103 | 12 | |
| Overwhelm | 1.21 | 0.0117 | 8 | |
| Safety Behaviours | 0.736 | 0.00749 | 0 | |

| ***Figure 4.*** Differences between centrality estimates: statistically significant differences indicated by a black square, non-significant differences by a grey square | | |
| --- | --- | --- |
| *Panel 4a: Strength (degree centrality)* 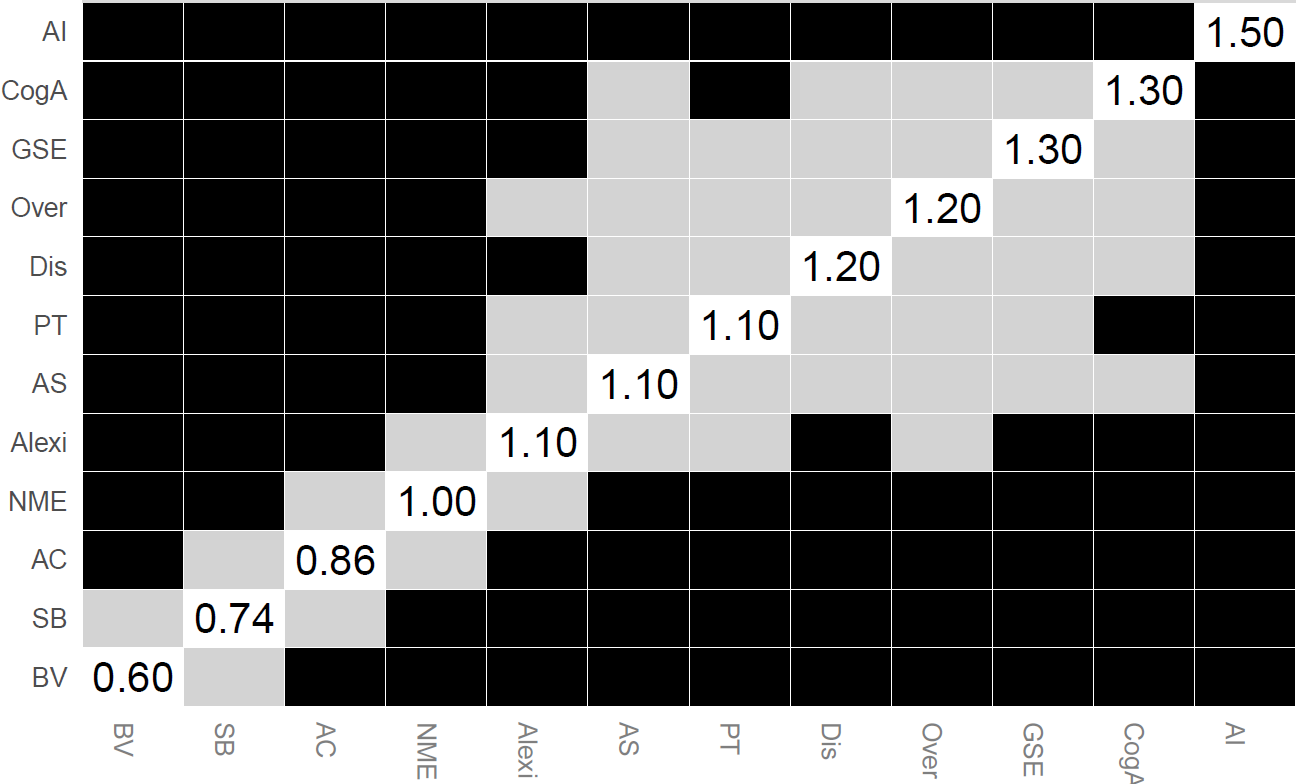 | *Panel 4c: Betweenness* 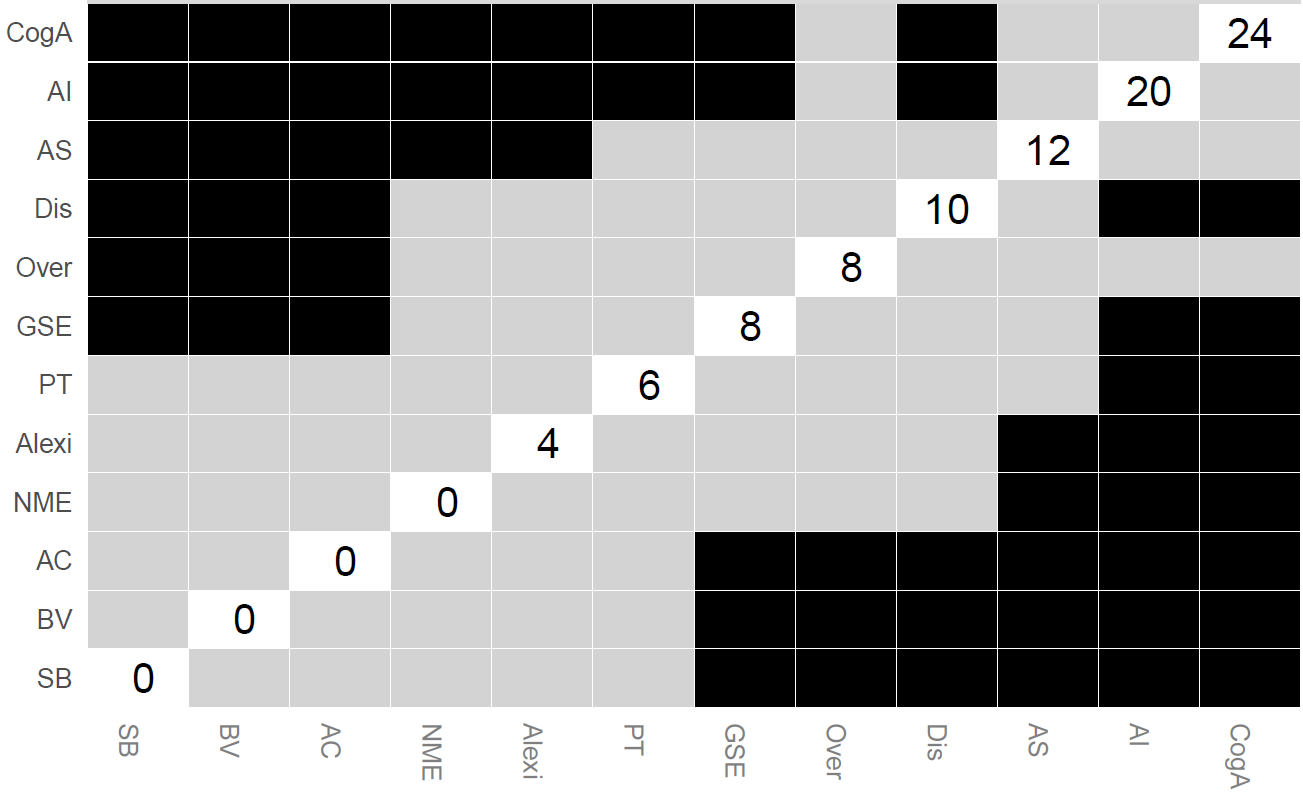 | |
| *Panel 4b: Closeness*  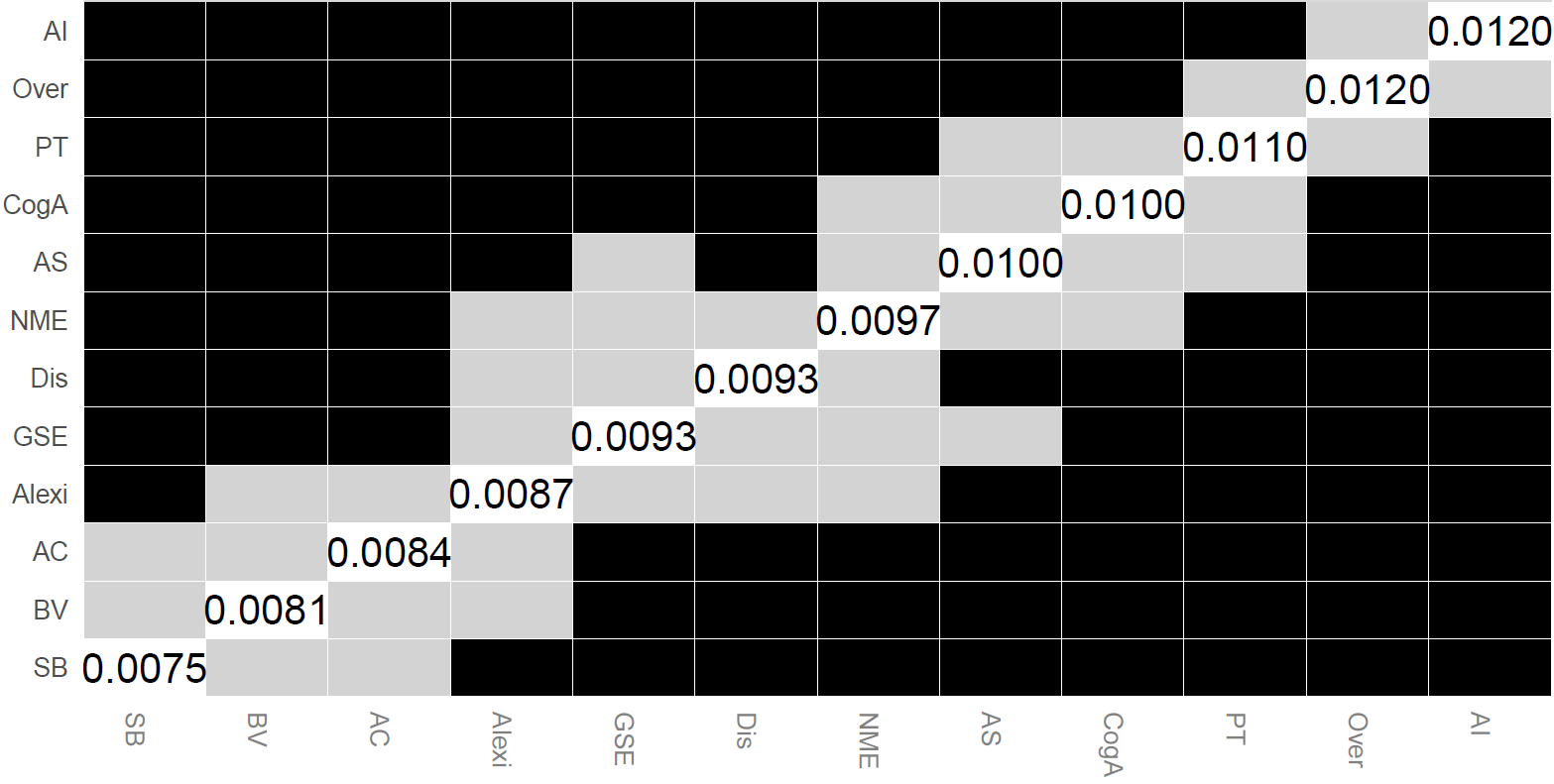 | | \| ***Key:*** \| \| \| --- \| --- \| \| *AC* \| *Attentional control* \| \| *AI* \| *Affect intolerance* \| \| *Alexi* \| *Alexithymia* \| \| *AS* \| *Anxiety sensitivity* \| \| *BV* \| *Body vigilance* \| \| *CogA* \| *Cognitive appraisals* \| \| *Dis* \| *Dissociation* \| \| *GSE* \| *General self-efficacy* \| \| *NME* \| *Negative meta-emotion* \| \| *Over* \| *Overwhelm* \| \| *PT* \| *Perseverative thinking* \| \| *SB* \| *Safety behaviours* \| |

The results of the centrality estimates indicate that affect intolerance has the highest node strength and closeness estimates in the network. Difference tests found that its node strength is significantly higher than any other variable and its closeness estimate significantly higher than for all other variables except overwhelm. The variable of cognitive appraisals was found to have the highest betweenness score. However, difference tests found that this was not significantly greater than those for affect intolerance, anxiety sensitivity, or overwhelm. Overall, these results suggest a high level of inter-connectivity within the network, with affect intolerance being particularly important in terms of direct and indirect pathways.

Finally, the stability of centrality estimates was calculated using case-dropping subset bootstrapping (Figure 5). This indicated that the stability for all three centrality measures was good. Correlation stability coefficients for betweenness was 0.59 and for closeness and strength were 0.75, which meets the recommendation that coefficients should be above 0.50.

***Figure 5.*** Showing the results of the case-drop subset bootstrapping


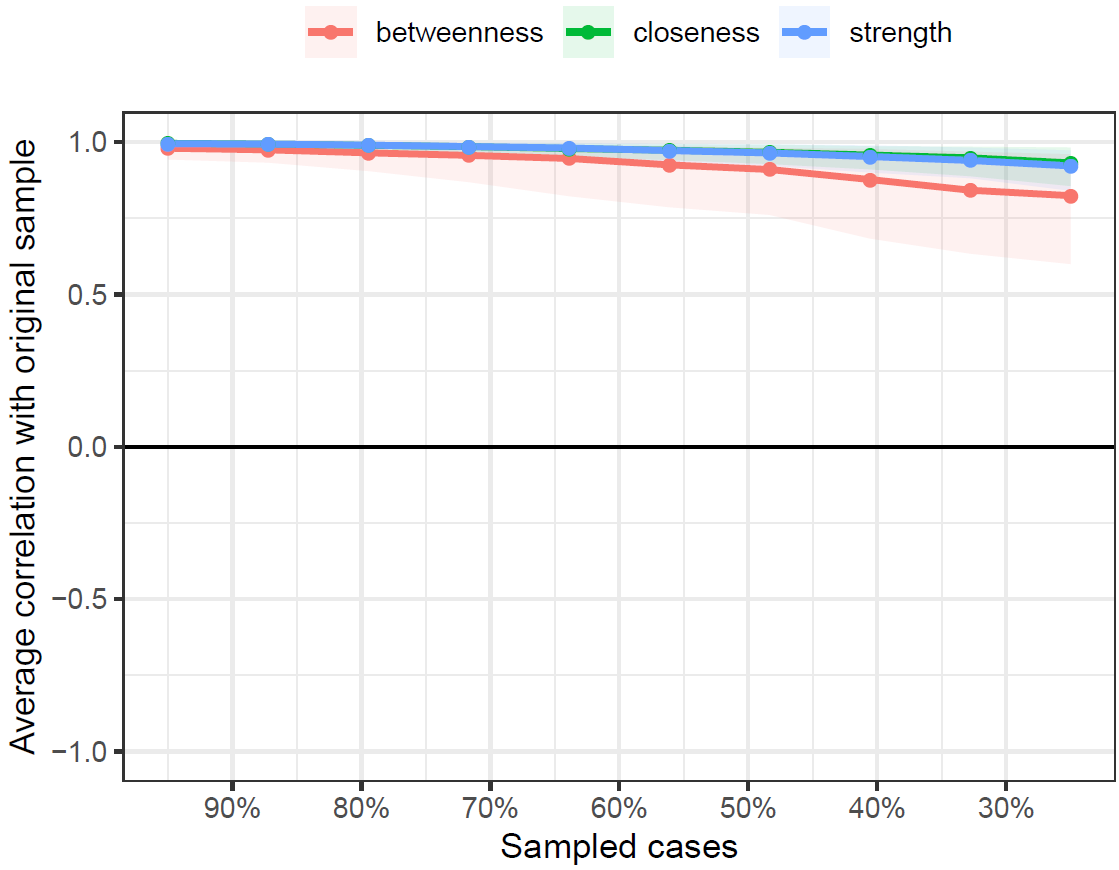

Supplement: Multimedia component 1 [file mmc1.docx]
